# Gut microbiota developmental trajectories in min and DLY pigs during lactation and nursery stages and the alleviation of ETEC-induced diarrhea by fecal microbiota transplantation

**DOI:** 10.3389/fmicb.2026.1939230

**Published:** 2026-10-26

**Authors:** Weichun Xie, Zhuoran Li, Jinru Lin, Huitao Bai, Jiaxuan Li, Wen Cui, Yanping Jiang, Baifen Song, Xiaona Wang, Lijie Tang

**Affiliations:** 1 College of Veterinary Medicine, Northeast Agricultural University, Harbin, China; 2 Key Laboratory of Dairy Science, Ministry of Education, Department of Food Science, Northeast Agricultural University, Harbin, China; 3 National Key Laboratory of Veterinary Public Health and Safety, College of Veterinary Medicine, China Agricultural University, Beijing, China; 4 Heilongjiang Key Laboratory for Animal Disease Control and Pharmaceutical Development, Northeast Agricultural University, Harbin, China

**Keywords:** Duroc × landrace × Yorkshire, Enterotoxigenic *Escherichia coli* K88, fecal microbiota transplantation, gut microbiota, min pig

## Abstract

**Introduction:**

The gut microbiota plays a vital role in porcine growth, intestinal barrier function, and disease resistance, yet the influence of host genetics on microbiota development and breed-specific phenotypes remains unclear. This study investigated gut microbiota dynamics in genetically distinct pig breeds and evaluated the functional impact of breed-specific microbiota on intestinal health.

**Methods:**

A total of 76 healthy 3-day-old piglets from Min pigs and Duroc × Landrace × Yorkshire (DLY) pigs were monitored from day 3 to day 49. Microbiota composition was analyzed at eight developmental stages using high-resolution 2bRAD-M sequencing. Intestinal tissues were collected at day 49 for spatial microbiota profiling and barrier function assessment. Additionally, twelve 21-day-old DLY piglets were assigned to control, Enterotoxigenic *Escherichia coli* (ETEC) K88 challenge, and fecal microbiota transplantation (FMT) groups to evaluate the protective effects of Min pig microbiota.

**Results:**

Significant breed-specific differences were observed in microbial composition, developmental trajectories, and ecological succession patterns. Min pigs exhibited higher microbial diversity and greater community stability during early weaning compared to DLY pigs, accompanied by improved intestinal barrier integrity and reduced diarrhea incidence. FMT from Min pigs significantly enhanced microbial diversity, strengthened intestinal barrier function, and alleviated diarrhea following ETEC challenge.

**Discussion:**

These findings demonstrate that host genetics shapes gut microbiota development, influencing intestinal health and disease resistance. Microbiota derived from Min pigs confers protective effects against enteric infection, highlighting its potential as a microbial resource to improve gut health in commercial pig production.

## 1 Introduction

Research increasingly highlights the significant role of gut microbiota in host immune defense (Kuziel and Rakoff-Nahoum, 2022). Gut microbiota dysbiosis is linked to various diseases such as inflammatory bowel disease, cardiovascular disease, allergies, diabetes, and obesity (Qin et al., 2012). The gut microbiota of animals changes with age, environment, stress, diet, health conditions, and antibiotic use (Cresci and Bawden, 2015). The development of the porcine gut microbiota, like that of other animal species, is influenced by age, with birth and weaning being pivotal life stages (Karasova et al., 2021; Shen et al., 2024).

In conventional pig farming, weaning occurs at 3 to 4 weeks, transitioning pigs from sow’s milk, rich in fat and lactose, to solid feeds with complex carbohydrates (van Nieuwamerongen et al., 2018). The weaning period is crucial in pig production, greatly affecting farm profitability (Ding et al., 2022). However, this transition involves sudden nutritional, physiological, and psychological stress, leading to weaning stress in pigs. This stress alters small intestinal morphology and function, impairs digestion and absorption capacity, disrupts the intestinal barrier, and triggers dysbiosis (Li et al., 2018; Tang et al., 2022). As a result, pigs experience decreased feed consumption, higher diarrhea incidence, and stunted growth.

Enterotoxigenic *Escherichia coli* (ETEC) infections are the primary cause of post-weaning diarrhea in pigs, leading to significant economic losses worldwide in the swine industry (Kim et al., 2022). ETEC adheres to the small intestine, initiating colonization and infection. Enterotoxin secretion compromises the intestinal barrier and triggers inflammation in weaned pigs (Yan et al., 2024). Probiotics can restore mucosal barrier function, balance intestinal flora, inhibit pathogen proliferation, and enhance both local and systemic immunity, thereby strengthening the intestinal barrier (Shen et al., 2018). FMT seeks to rapidly reestablish the normal function and composition of the gut microbiota, aiding in the treatment of both intestinal and extraintestinal diseases. FMT has shown potential as an effective treatment for multiple diseases (Li et al., 2022). FMT acts as a strategy to protect against ETEC colonization and infection.

Growing evidence suggests that gut microbiota composition differs significantly between indigenous Min pigs and commercial Duroc × Landrace × Yorkshire (DLY) pigs, potentially influencing their health and disease resistance. However, the dynamics of microbial colonization, development, and maturation across intestinal segments remain poorly characterized. 2bRAD-M is a streamlined genome-wide methylation sequencing method utilizing type IIB restriction enzymes, facilitating concurrent examination of gut microbiota composition and DNA methylation patterns for microbial epigenetics research (Yi et al., 2024).

Furthermore, whether these microbial differences contribute to enhanced gut barrier function and resilience to pathogens, such as ETEC K88-requires further investigation. This study explores how FMT from Min pigs to DLY pigs modulates gut microbial ecology, intestinal barrier integrity, and resistance to ETEC infection. Our findings provide insights into host-microbiome interactions and may aid in identifying beneficial microbial candidates for improving swine health and reducing antibiotic reliance.

## 2 Materials and methods

### 2.1 Animal ethics statement

The Animal Ethics Committee of Northeast Agriculture University approved this study, with the approval code NEAUEC20220317.

### 2.2 Animals and experimental design

A total of 76 healthy 3-day-old piglets, with an average body weight of 1.2 ± 0.75 kg and belonging to two breeds (DLY pigs and Min pigs), were randomly selected from the offspring of different sows at Harbin Gushi Agricultural and Animal Husbandry Group Co., Ltd. and Lanxi Breeding Farm (Lanxi, Heilongjiang, China), and all piglets were ear-tagged for identification. Piglets were kept on a 12-h dark–light cycle with unrestricted access to food and water. The room temperature was kept at 30 °C. The experimental diet was a corn-soybean meal-based basal diet, and its composition and nutritional levels are shown in Supplementary Table S1. All diets were autoclaved to eliminate exogenous microbial contamination. During the experiment, the mental status, feeding behavior, and fecal characteristics of piglets were observed daily, with any abnormalities recorded and addressed promptly. Prior to tissue and blood sampling, all piglets were fasted overnight. Each piglet was humanely euthanized via intramuscular administration of Zoletil 50 (tiletamine-zolazepam mixture, Beijing Lab Anim Tech Develop Co., Ltd., Beijing, China) at a standardized dose of 15 mg/kg body weight. Complete loss of corneal reflex, disappearance of spontaneous breathing, and cardiac arrest were jointly verified to confirm irreversible death before any sample collection procedures were carried out. The animal experiments were divided into three distinct sections as follows.

### 2.3 Comparative experiment on gut microbiota between min pigs and DLY pigs

Following the principles of similar body weight and equal numbers of males and females, a total of 8 piglets, including both DLY pigs and Min pigs, were selected. The experiment adopted an individual feeding system. Piglets were fed three times daily at 07:00, 13:00, and 19:00.

Under consistent feeding conditions, anal swab samples were collected from pigs at eight developmental stages (days 3, 7, 14, 21, 28, 35, 42, and 49). For in-depth intestinal tissue analysis, sample pigs were screened based on the criteria that their ADFI and average daily gain (ADG) were within the range of mean ± 10% of the group. Statistical verification confirmed no significant difference between the selected pigs and the whole group (*p* > 0.05), ensuring the representativeness of the samples. At least four anal swab samples were obtained from each selected pig. After the final sampling, all selected pigs were fasted for 8 h and then humanely euthanized. The entire intestinal tissue was excised and segmented into the duodenum, jejunum, ileum, cecum, and colon. Each intestinal segment was cut open with sterilized scissors, and the contents were collected into 2 mL freezing tubes. These samples were promptly snap-frozen in liquid nitrogen and stored at −80 °C for subsequent analysis.

### 2.4 Analysis of intestinal mucosal barrier function between min pig and DLY pig

The weight changes, survival rate, and incidence of diarrhea in Min Pigs and DLY Pigs were monitored over a 49-day period. At day 49, all pigs were humanely euthanized. Intestinal samples (duodenum, jejunum, and ileum) were collected for various analyses. The sample pigs were selected based on the consistency of their growth performance with the group average, and no significant statistical difference was found (*p* > 0.05).

### 2.5 FMT and ETEC infection experiment

Fresh feces were collected before weaning, and the collected feces were put into a sterile container and filtered through tools such as gauze or strainer to remove food residues, hair and other impurities from them. An appropriate amount of sterile saline or another specific buffer solution was added to the filtered feces, which was then mixed thoroughly using a homogenizer to make a fecal homogenate that stored at −80 °C.

Twelve 21-day-old DLY piglets with comparable body weight and litter origin were randomly assigned to three groups (*n* = 4 each): a normal control group, an ETEC-infected group, and an FMT-treated group (ETEC+FMT). The basal diet composition and nutritional levels are presented in Supplementary Table S1. All piglets were housed individually and fed three times daily at fixed times. The FMT-treated group received daily oral administration of fecal microbiota suspension (2 mL/pig/day) for 5 consecutive days, while the other two groups were given an equal volume of sterile normal saline. Anal swab samples were collected at days 28 and 42. After sample collection on day 42, piglets in both the ETEC-infected and FMT-treated groups were orally challenged with the ETEC K88 strain (6 × 10^9^ CFU/mL, 20 mL/pig/day) for 5 consecutive days, whereas the control group continued to receive sterile normal saline. For sample collection, piglets in each group were selected according to the criteria that their ADFI and body weight gain were consistent with the group average, and the representativeness was verified by statistical analysis (*p* > 0.05). The sample collection and processing methods were consistent with the aforementioned protocols.

### 2.6 DNA extraction and sequencing

DNA (1 pg-200 ng) was digested with BcgI enzyme (4 U, NEB) for 3 h at 37 °C, then ligated with adaptors using 10 μL of digested DNA, 10 μL of ligation mix (0.2 μM adaptors, 800 U T4 ligase), and incubated at 4 °C for 12 h. The ligation products were amplified and analyzed on an 8% polyacrylamide gel. Bands around 100 bp were excised and incubated in nuclease-free water for 6–12 h. Sample barcodes were added by PCR with primers containing platform-specific barcodes. The PCR mix (20 μL) included 6 μL of gel-extracted product, primers, dNTPs, Phusion buffer, and Phusion polymerase (NEB). PCR products were purified (QIAquick kit, Qiagen) and sequenced on the Illumina Nova PE150 platform. 2bRAD-M analysis was conducted at Qingdao OE Biotech Co., Ltd. (Qingdao, China).

### 2.7 Data analysis

A total of 404,199 microbial genomes (bacteria, fungi, archaea) from the GTDB and Ensembl databases were used to create a 2bRAD marker database. Perl scripts extracted restriction fragments using 16 type 2B enzymes, and each genome’s 2bRAD tags were linked to its taxonomic information (Sun et al., 2022; Sun et al., 2023).

After quality control, sequenced tags were mapped to the database, which includes unique 2bRAD markers for 86,022 microbial species. To minimize false positives in species identification, a G score was calculated based on read coverage and the number of potential markers. A threshold of 5 was set to reduce false-positive identifications. Statistical analyses were performed using R software. Paired Wilcoxon tests were applied to compare the α-diversity of different groups based on chao 1 and Shannon indices, which reflect species richness and evenness, respectively. Principal coordinate analysis (PCoA) based on Binary Jaccard and Bray-Curtis distances was conducted to evaluate the differences in β-diversity among groups. Additionally, PICRUSt2 software was used to predict the potential functions of microorganisms across different developmental stages.

### 2.8 Animal performance and diarrheal score

Pig weights were recorded weekly throughout the experiment, and the average daily weight gain was calculated on days 3, 7, 14, 21, 28, 35, 42, and 49. The health and diarrhea incidence in pigs infected with ETEC K88 were monitored daily, and the diarrhea rate was determined.

Samples were collected on day 49 following euthanasia. The fecal score was assessed using a 5-point scale based on fecal appearance: 0 for normal, 1 for loose stool, 2 for loose/some diarrhea, 3 for diarrhea, and 4 for severe watery diarrhea. Diarrhea was considered when the fecal score exceeded 2. Formula: Diarrhea rate (%) = number of pigs with diarrhea during the test period / (number of test days × number of pigs in the same group) × 100% (Xie et al., 2021).

### 2.9 Intestinal morphology measurement

Jejunum sections were fixed in 4% paraformaldehyde, dehydrated in a graded ethanol series, embedded in paraffin, and sectioned at 2 μm thickness. The sections were stained with hematoxylin and eosin (H&E) and alcian blue PAS (AB-PAS). Villus height and crypt depth were measured at 40 × magnification (Olympus BX43, Tokyo, Japan). Villus height and crypt depth were measured using ImageJ software (National Institutes of Health, Bethesda, MD, USA). Images were spatially calibrated using the microscope scale before measurement. Only well-oriented villus-crypt units with an intact epithelial structure were included. Villus height was measured from the villus tip to the villus-crypt junction, whereas crypt depth was measured from the villus-crypt junction to the base of the crypt. Five representative villus-crypt units were measured for each section. Measurements were performed manually by the same trained investigator.

### 2.10 Real-time quantitative PCR

RNA was extracted by TRIZOL method. Total RNA concentrations were determined using a Nano-Drop 2000 spectrophotometer. A ReverTra Ace qPCR RT Kit (Toyobo) was used for RT. Target gene expression changes were analyzed using the 2^−ΔΔCt^ method, with samples normalized to GAPDH. The primers are shown in Supplementary Table S2.

### 2.11 Western blotting

Total protein from intestinal mucosal tissues was extracted using RIPA buffer (Beyotime, China) and quantified with a BCA protein assay kit (Beyotime, China). Equal amounts of protein were separated by 10% SDS-PAGE (Seven, China) and transferred to PVDF membranes. Membranes were blocked with 5% non-fat milk for 1 h at room temperature and then incubated overnight at 4 °C with primary antibodies against Claudin-1 and Occludin. After washing with PBST, membranes were incubated with HRP-conjugated secondary antibodies for 2 h at room temperature. Mix solution A and solution B of Super ECL Plus Ultra-Sensitive Luminescent Reagent at a 1:1 ratio, add the mixture evenly onto the membrane for color development, and then perform exposure and imaging.

### 2.12 Determination of the amount of ETEC K88

Mucus samples from each intestinal segment were processed with a bacterial genome extraction kit. After resuspending the supernatants in an equal volume of PBS, they were centrifuged, and total bacterial DNA was extracted following the kit’s protocols. Subsequently, the gene copy number of ETEC K88 in each intestinal segment was quantitatively analyzed by real-time fluorescence quantitative PCR using primers specific for ETEC K88.

### 2.13 Scanning electron microscope (SEM)

Following treatment, the segment was gently washed with PBS. After a 2-h fixation at room temperature, move the petri dish to 4 °C. The tissue blocks were then rinse tissue blocks three times with 0.1 M PBS, allowing 15 min for each wash. Subsequently, immerse tissue blocks in 1% OsO4 prepared in 0.1 M PBS for 1–2 h at room temperature. Subsequently, rinse the segment blocks three times in 0.1 M PBS, allowing 15 min for each wash. The segments were sequentially washed with increasing concentrations of ethanol: 30%, 50%, 70%, 80%, 90%, and 95%, each for 15 min. Two 15-min changes of 100% ethanol were performed. The samples were subsequently placed in isoamyl acetate for 15 min. Specimens were mounted on metallic stubs using carbon stickers and sputter-coated with gold for 30 s before being imaged with a scanning electron microscope.

### 2.14 Analysis of pig serum for inflammatory cytokines and immunoglobulins

Blood samples were collected from the anterior vena cava into serum collection tubes before euthanasia. Samples were allowed to clot at room temperature for 30 min and centrifuged at 3000 × *g* for 15 min at 4 °C. The resulting serum was collected and stored at −80 °C until analysis. Serum IL-6 and TNF-α levels were quantified using ELISA kits (Jiangsu Enzyme-Labeled BioTECH, Yancheng, China) in accordance with the manufacturer’s protocol.

### 2.15 Statistical analysis

Experimental data were plotted using GraphPad Prism 9.0. Multivariate statistical analysis was performed via one-way analysis of variance (ANOVA), followed by Tukey’s *post-hoc* test for comparisons between groups. The grayscale values of protein bands from Western blot assays were quantitatively analyzed using ImageJ software. Statistical significance was considered at *p* < 0.05. Where applicable, statistical significance is indicated as *p < 0.05, **p < 0.01, and ***p < 0.001. Different symbols used for specific pairwise comparisons are defined in the corresponding figure legends.

## 3 Results

### 3.1 Dynamic succession of gut microbiota during lactation and nursery periods in DLY pigs

We examined the longitudinal alterations in the gut microbiota of DLY pigs during three crucial developmental stages: the lactation period (days 3, 7, 14, 21, and 28 post-birth), the early weaning phase (day 35), and the later stage (days 42 and 49). Microbial alpha diversity, assessed via the chao1 and Shannon indices (Figures 1A,B), exhibited a progressive increase with age. Notably, a sharp decline occurred at day 35, corresponding to weaning-followed by a recovery at day 42. Post-weaning, alpha diversity remained relatively stable despite the introduction of solid feed.

**Figure 1:**
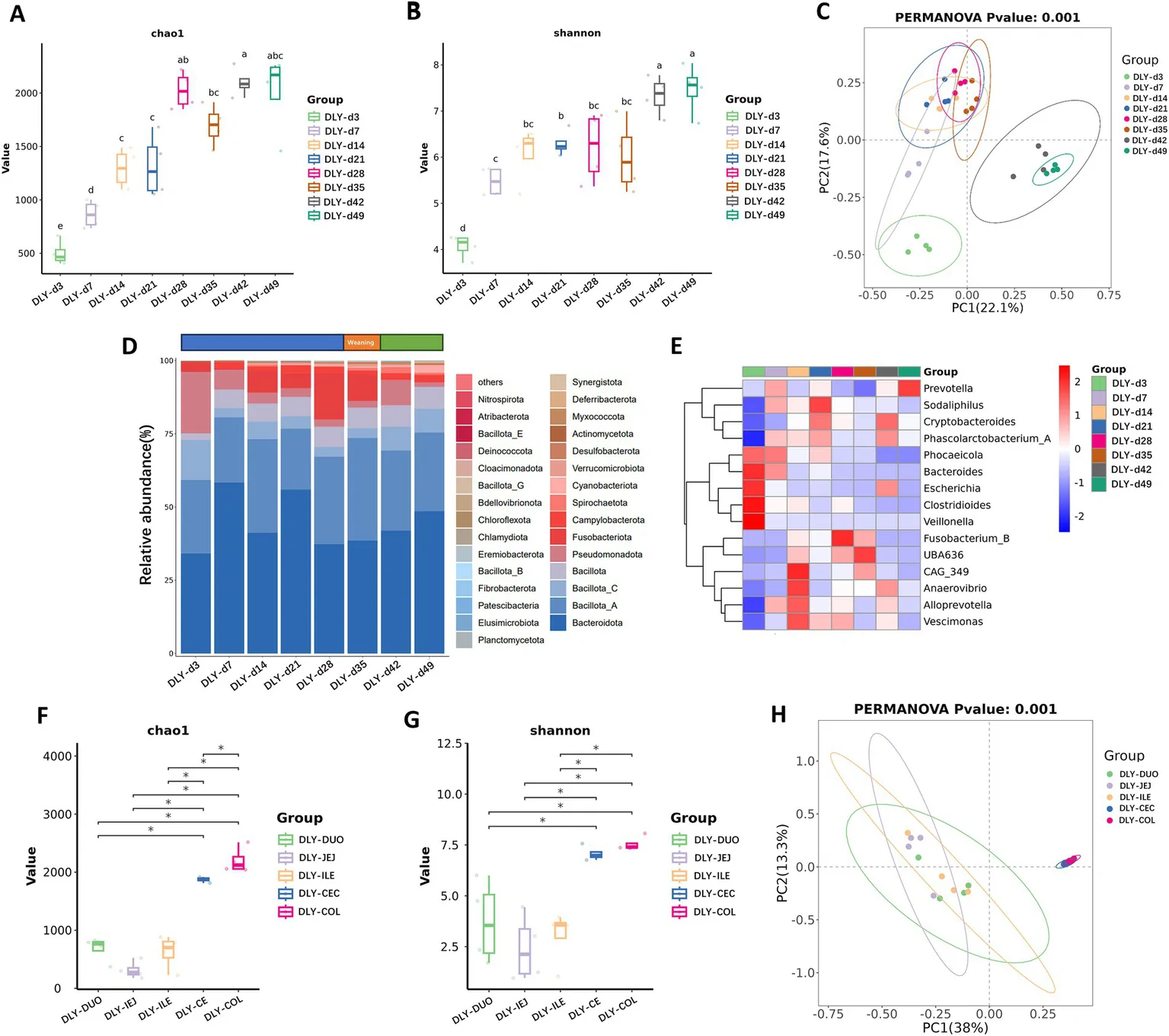
Gut microbiota analysis of DLY pigs. **(A)** α-diversity analysis at different time points in DLY pigs (chao1). **(B)** α-diversity analysis at different time points in DLY pigs (Shannon). **(C)** β-diversity analysis at different time points in DLY pigs (PCoA). **(D)** Bar chart of gut microbiota composition at the phylum level. **(E)** Heatmap of gut microbiota composition at the genus level. **(F)** α-diversity analysis of each intestinal segment in DLY pigs (chao1). **(G)** α-diversity analysis of each intestinal segment in DLY pigs (Shannon). **(H)** β-diversity analysis of each intestinal segment in DLY pigs (PCoA). Different lowercase letters (a, b, c, d, e) indicate statistically significant differences between groups (*p* < 0.05), while the same letter indicates no significant difference. Statistical significance denoted as follows: *p < 0.05; **p < 0.01; ***p < 0.001; ns *p* > 0.05.

We conducted PCoA using the binary Jaccard distance to further investigate community structure (Figure 1C). An Analysis of Similarities (ANOSIM) using unweighted UniFrac distances identified significant compositional differences between nursing and weaned pigs, especially on day 3. Comparative analyses of microbial composition at the phylum and genus levels are shown in Figure 1D and Figure 1E. *Bacteroidota* and *Bacillota* were the predominant phyla in the gut microbiota, comprising over 70% of the total sequences. The heatmap in Figure 1E highlights the 30 most abundant bacterial genera, with *Prevotella* emerging as the most prevalent, particularly increasing after weaning. In contrast, the abundance of *Bacteroides* gradually declined with age. During early weaning, the chao1 and Shannon indices (Figures 1F,G) across intestinal segments on day 49 showed that microbial diversity and relative abundance were greater in the large intestine (cecum, colon) compared to the small intestine (duodenum, jejunum, ileum). PCoA analysis showed clear differences in microbial composition among intestinal segments (day 49) during early weaning. Cecal and colonic samples clustered closely, indicating similar communities, while duodenal, jejunal, and ileal samples also showed high similarity. In contrast, small- and large-intestine samples were clearly separated, suggesting distinct microbial structures between these regions in DLY pigs (Figure 1H).

### 3.2 Dynamic succession of gut microbiota during lactation and nursery periods in min pigs

We analyzed gut microbiota diversity and abundance at the genus and species levels, as well as the total number of probiotics across developmental time points. The results showed increased microbiota diversity and abundance post-weaning, although at a slower rate than during the pre-weaning period (Figures 2A,B). PCoA analysis revealed a distinct separation between the gut microbiota profiles of nursing and weaned pigs (Figure 2C), indicating substantial compositional shifts from birth through weaning.

**Figure 2:**
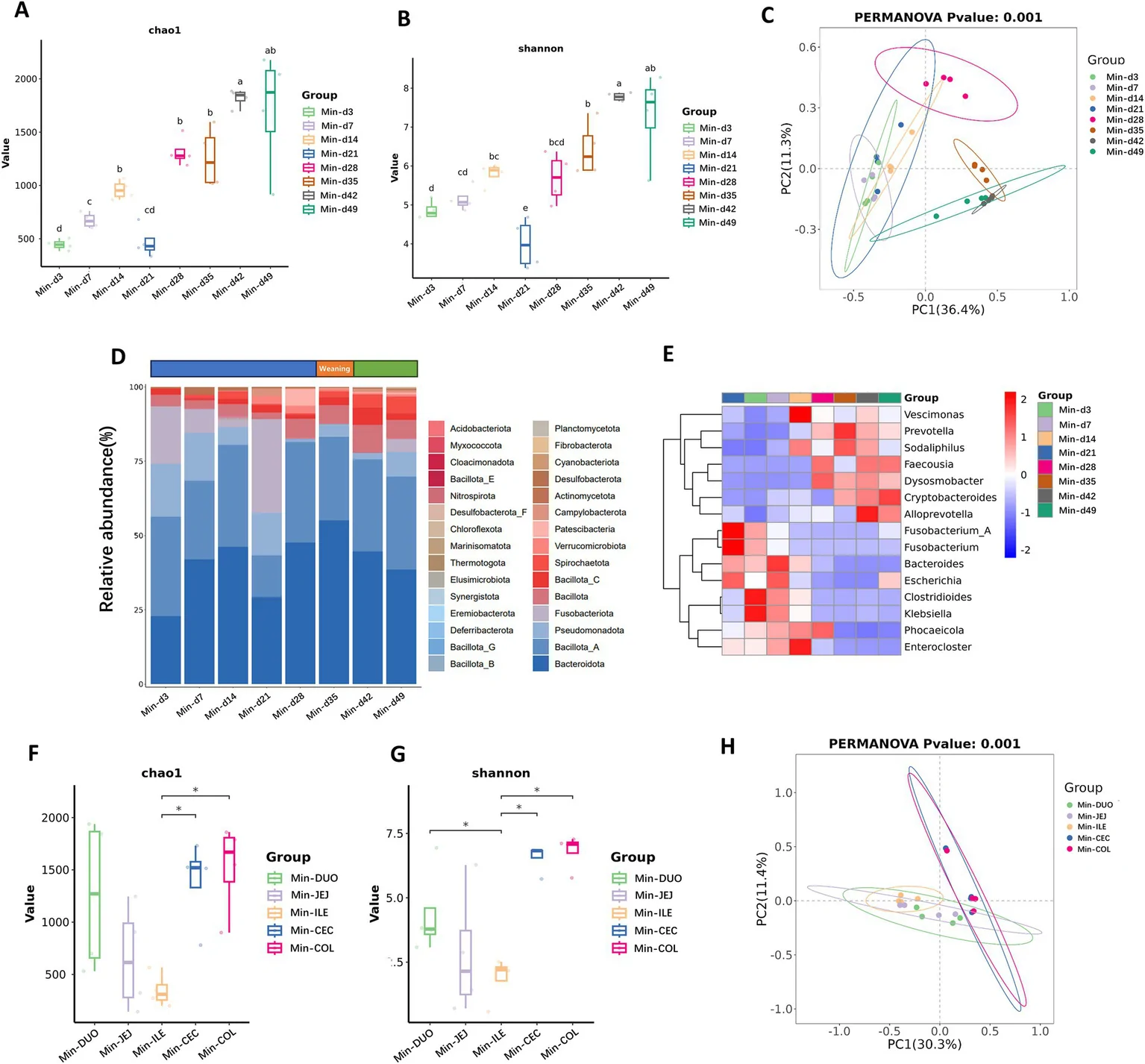
Gut microbiota analysis of Min pigs. **(A)** α-diversity analysis at different time points in Min pigs (chao1). **(B)** α-diversity analysis at different time points in Min pigs (Shannon). **(C)** β-diversity analysis at different time points in Min pigs (PCoA). **(D)** Bar chart of gut microbiota composition at the phylum level. **(E)** Heatmap of gut microbiota composition at the genus level. **(F)** α-diversity analysis of each intestinal segment in Min pigs (chao1). **(G)** α-diversity analysis of each intestinal segment in Min pigs (Shannon). **(H)** β-diversity analysis of each intestinal segment in Min pigs (PCoA). Different lowercase letters (a, b, c, d, e) indicate statistically significant differences between groups (*p* < 0.05), while the same letter indicates no significant difference. Statistical significance denoted as follows: *p < 0.05; **p < 0.01; ***p < 0.001; ns *p* > 0.05.

To explore microbial colonization dynamics, we examined the sequence and timing of bacterial taxa emergence from birth to 2 weeks post-weaning. At the phylum level, the top 30 phyla were identified, with *Bacteroidota*, *Pseudomonadota*, *Bacillota*, and *Fusobacteriota* being dominant. Among them, *Bacteroidota* was the most abundant, followed by *Bacillota*, and together they accounted for approximately 70% of total sequences across all developmental stages (Figure 2D). A heatmap was generated to display the 15 most abundant genera at the genus level. *Prevotella* emerged as the most diverse and dominant genus during the study, especially following the introduction of solid feed. Notably, *Cryptobacteroides* emerged as the predominant bacterial genus in Min pigs during both post-weaning time points (Figure 2E). At early weaning (day 49), α-diversity varied across intestinal segments. The chao1 and Shannon indices were higher in the cecum and colon compared to the ileum, while the duodenum showed a different Shannon index than the ileum (Figures 2F,G). PCoA (Figure 2H) revealed comparable microbial compositions within the small and large intestines, yet distinct separation between them, indicating unique communities in the Min group, aligning with the DLY group.

### 3.3 Pig breed determines divergent development of gut microbiota composition and diversity

Significant variations were observed in the gut microbiota composition and intestinal traits among different pig breeds. After weaning, the diversity and abundance of gut microbiota in Min pigs increased again, though at a slower rate than during the pre-weaning period, whereas the microbial community in DLY pigs remained relatively stable after 28 days of age. Alpha diversity analysis of fecal microbiota (Figures 3A,B) revealed that microbial richness in both pig breeds initially declined before increasing. Nursing DLY piglets exhibited high microbial richness up to day 28, followed by a marked reduction in alpha diversity by day 35 post-weaning. Notable differences in gut microbiota composition were observed between the two breeds during the early-life and weaning stages, with Min pigs showing greater abundance of probiotic species such as *Bacteroides* and *Prevotella* compared to DLY pigs (Supplementary material S1). To explore regional variation in gut microbiota, we analyzed microbial communities from different intestinal segments-including both large (cecum and colon) and small (duodenum, jejunum, and ileum) intestines-at 49 days of age. Alpha diversity analysis (Figures 3C,D) indicated that the large intestine harbored greater microbial diversity and richness than the small intestine. Beta diversity analysis (Figure 3E) revealed distinct clustering of microbiota from Min and DLY pigs. Non-metric multidimensional scaling (NMDS) based on Bray-Curtis distances demonstrated significant compositional variation between the two breeds within each intestinal segment (Figure 3F).

**Figure 3:**
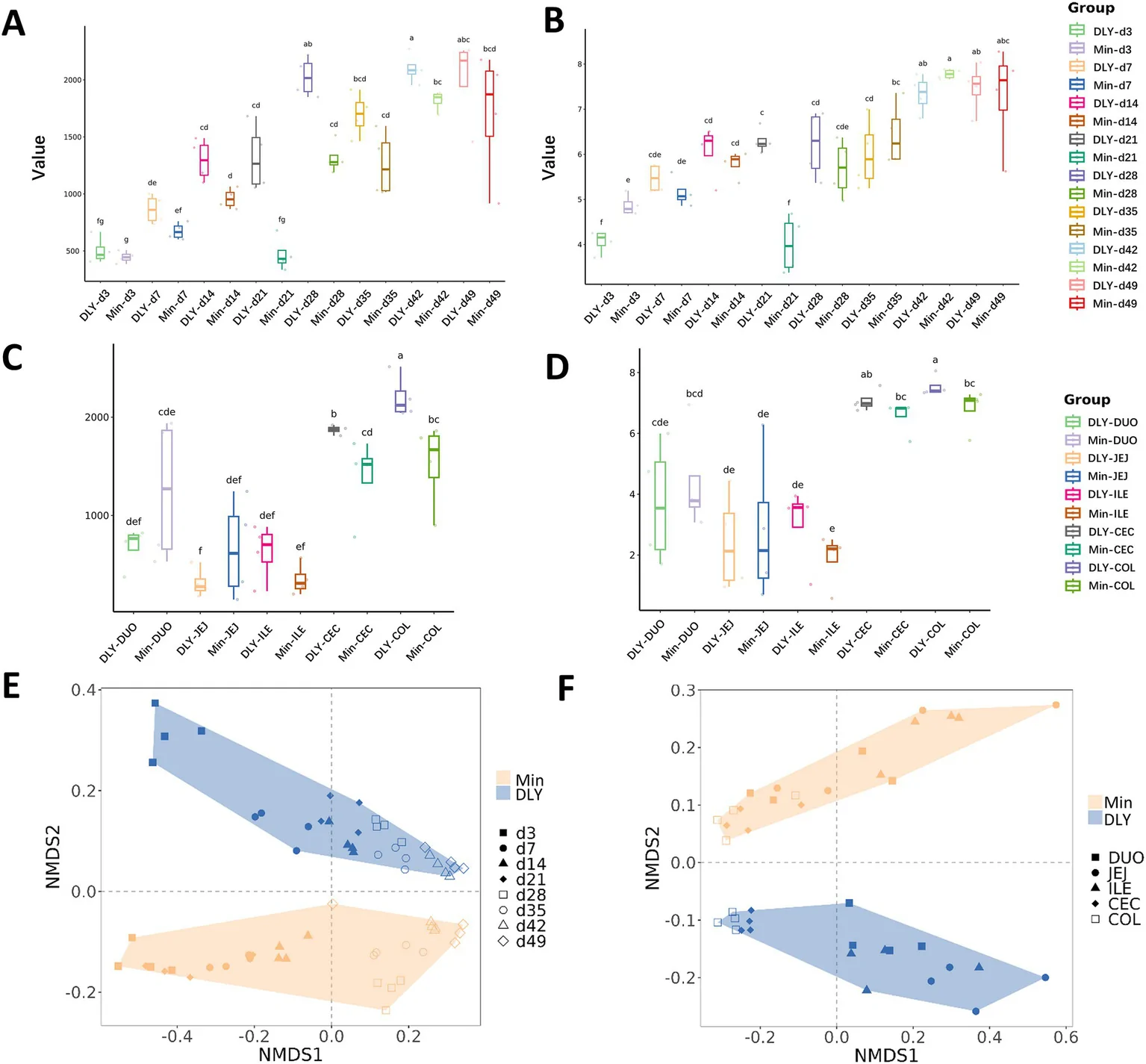
Diversity of gut microbiota of piglets at different stages and different intestinal segments. **(A)** α-diversity analysis at different time points (Chao1). **(B)** α-diversity analysis at different time points (Shannon). **(C)** α-diversity analysis of each intestinal segment (Chao1). **(D)** α-diversity analysis of each intestinal segment (Shannon). **(E)** β-diversity analysis at different time points. **(F)** β-diversity analysis of each intestinal segment. Different lowercase letters (a, b, c, d, e) indicate statistically significant differences between groups (*p* < 0.05), while the same letter indicates no significant difference.

Regarding fungal communities, fungal communities identified in DLY pigs postnatally included *Ascomycota*, *Microsporidia*, and *Cryptomycota*. Six fungal species were detected, with *Enterocytozoonidae*, *Saccharomycetaceae*, *Cordycipitaceae* and *Pichiaceae* before weaning. After weaning, *Hypocreaceae* became the dominant fungal species (Supplementary material S2A). *Parasitella* and *Paramicrosporidium* saccamoebae were the most prevalent fungal species in Min pigs, maintaining high abundance. Interestingly, no detectable gut fungal communities were observed in Min pigs at the time of birth (Supplementary material S2B).

### 3.4 Gut microbiota composition exhibits breed-specific differences in DLY and min pigs

At day 49, gut microbiota composition differed markedly across intestinal segments in both DLY and Min pigs (Figures 4A,B). At the phylum level, the small intestine was dominated by *Bacillota* and *Pseudomonadota*, whereas the large intestine was primarily composed of *Bacteroidota* and *Bacillota*. These segment-specific patterns were conserved across both breeds, indicating a strong anatomical influence on microbial distribution.

**Figure 4:**
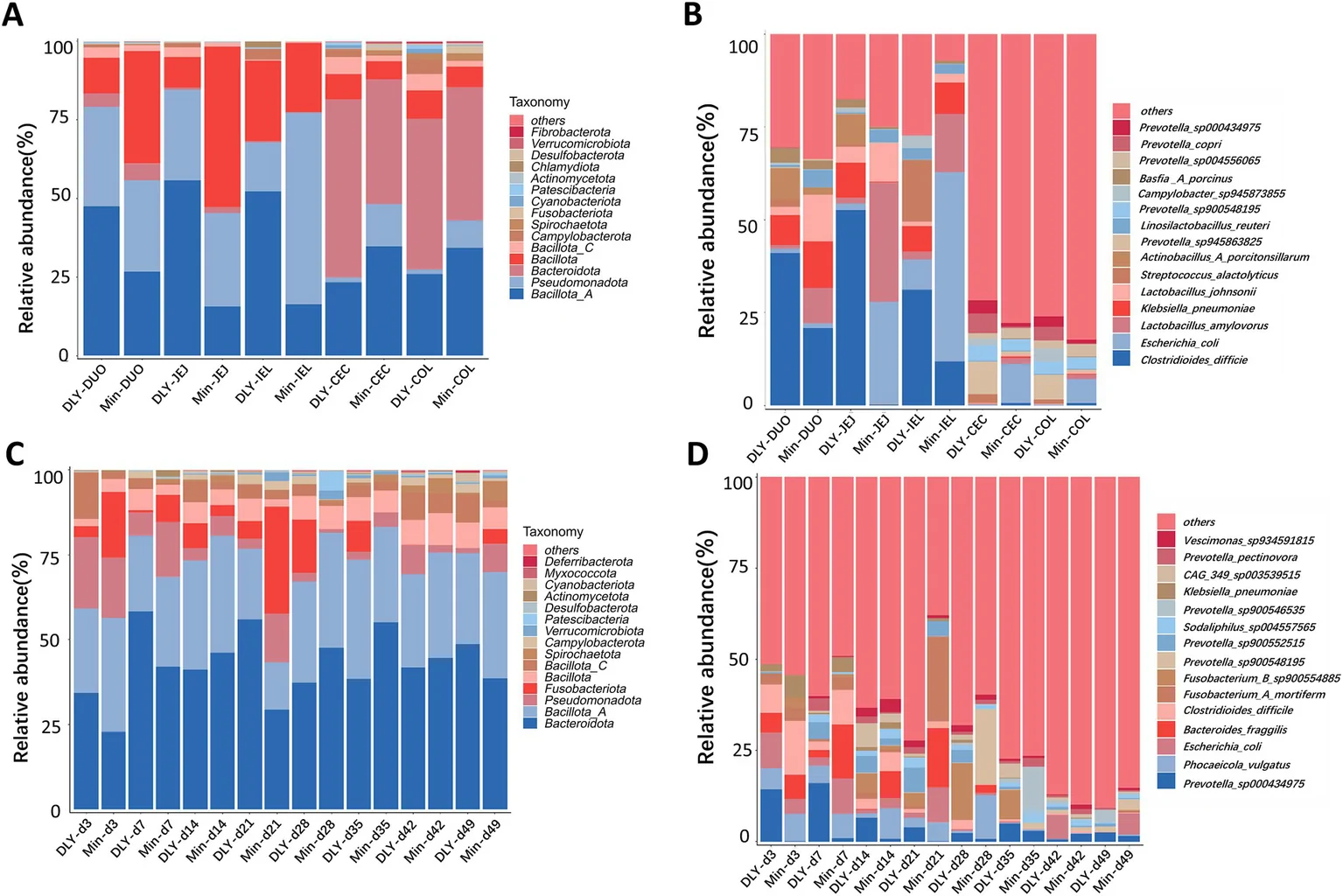
Segmental and age-dependent variations in gut microbial composition at phylum and species levels. **(A,B)** Relative abundance of gut microbiota composition at the phylum and species levels across different intestinal segments (day 49). **(C,D)** Relative abundance of gut microbiota composition at the phylum and species levels at different developmental time points.

Despite these shared spatial patterns, pronounced breed-specific differences were observed. In the small intestine, Bacillota A accounted for nearly 50% of the bacterial community in DLY pigs but only 20% in Min pigs. At the genus level, a total of 1,640 genera were annotated. Prevotella was predominantly enriched in the cecum and colon, while Escherichia and Lactobacillus were significantly more abundant in Min pigs than in DLY pigs. In contrast, Clostridioides dominated the gut microbiota of DLY pigs, accounting for approximately 41% of total bacterial abundance, which was significantly higher than in Min pigs (Figures 4A,B). SparCC network analysis further revealed distinct bacterial interaction patterns associated with different developmental stages (Supplementary material S3).

The temporal dynamics of gut microbiota development also differed between the two breeds (Figures 4C,D). At the phylum level, *Bacteroidota* and *Firmicutes* were dominant in both breeds, with no significant difference in overall abundance. However, *Fusobacteriota* was significantly enriched in Min pigs at days 3 and 7. At the species level, *Prevotella sp000434975* was more abundant in DLY pigs, whereas *Phocaeicola vulgatus* and *Bacteroides fragilis* were preferentially enriched in Min pigs. Notably, *Escherichia coli* showed significantly higher abundance in DLY pigs during both the neonatal and weaning periods.

Gut fungal communities also exhibited breed-specific characteristics. 2bRAD-M profiling identified five fungal phyla and 30 genera, with Min pigs displaying higher fungal diversity than DLY pigs (Supplementary materials S4A,B). In DLY pigs, fungal communities were mainly composed of *Basidiomycota*, *Microsporidia*, and *Ascomycota*, with the large intestine exclusively dominated by *Ascomycota*. In contrast, the large intestine of Min pigs was predominantly enriched in *Mucoromycota* and *Cryptomycota*. *Candida albicans* was the dominant fungal species in the large intestine of both breeds and accounted for approximately 47% of the fungal community in the duodenum of DLY pigs. In the small intestine, *Vanderwaltozyma polyspora*, *Purpureocillium lilacinum*, and *Saccharomyces cerevisiae* together constituted nearly 30% of the fungal community.

### 3.5 Gut microbiota of min pigs demonstrates superior metabolic capacity compared to DLY pigs

The Kyoto Encyclopedia of Genes and Genomes (KEGG) pathway analysis, utilizing PICRUSt, was performed to predict the functional profiles of gut microbiota in Min and DLY pigs. Min pigs showed greater capability than DLY pigs in terms of global and overview map, carbohydrate metabolism, metabolism, and other abilities, biosynthesis of secondary metabolites, and other bacterial microbiota functions (Figure 5A). COG pathway enrichment analysis revealed that both DLY and Min pigs experienced changes in gut microbiota functions as they aged. In Min pigs, key functional pathways such as metabolism, genetic information processing, environmental information processing, cellular processing, and organismal systems remained active up to 14 days post-birth. In contrast, DLY pigs showed a decline in microbiota functions during the first week of life (Figure 5B).

**Figure 5:**
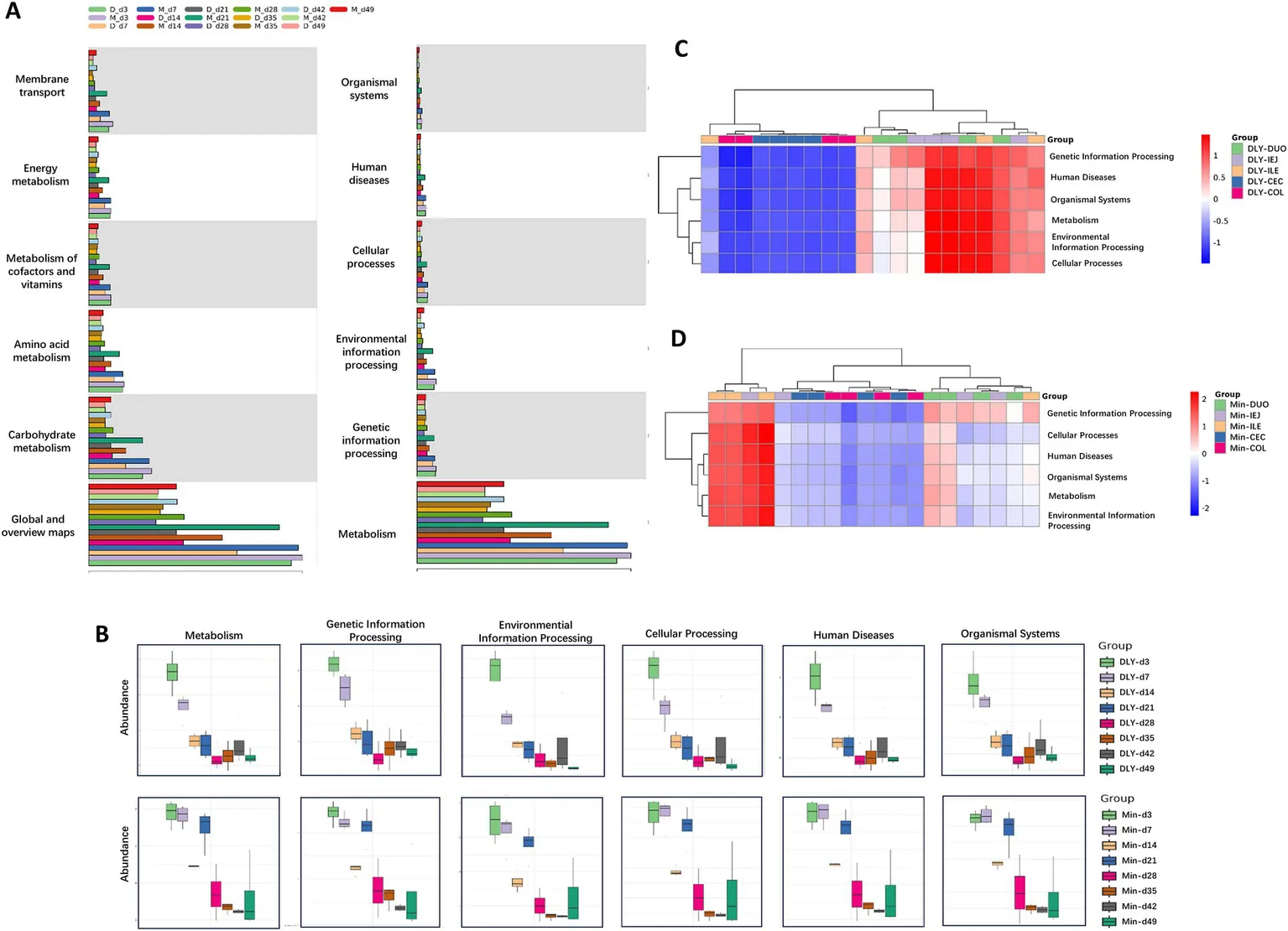
Functional analysis of gut microbiota. **(A)** KEGG level functional annotation abundance map. **(B)** COG pathway enrichment analysis of gut microbiota in DLY pigs and Min pigs. **(C,D)** Clustered heatmap of functional differences among intestinal segments (day 49). Blue indicates low relative abundance of COG and red indicates high relative abundance of COG.

In addition, we compared the functional profiles of gut microbiota in different intestinal segments (day 49) between DLY and Min pigs (Figures 5C,D). Compared to the cecum and colon, small bowel gut bacteria exhibited greater activity in metabolism, genetic information processing, environmental information processing, cellular processing, and organismal systems.

### 3.6 Min pigs outperform DLY pigs in gut resilience and anti-inflammation

Min pigs grew more slowly than DLY pigs but maintained steady body weight gain throughout the 49-day observation period. After weaning, DLY pigs exhibited a higher diarrhea rate than Min pigs, with the average diarrhea rate being approximately 25% lower in Min pigs (Figure 6A). These findings suggest that, despite superior growth, DLY pigs may be more susceptible to post-weaning diarrhea.

**Figure 6:**
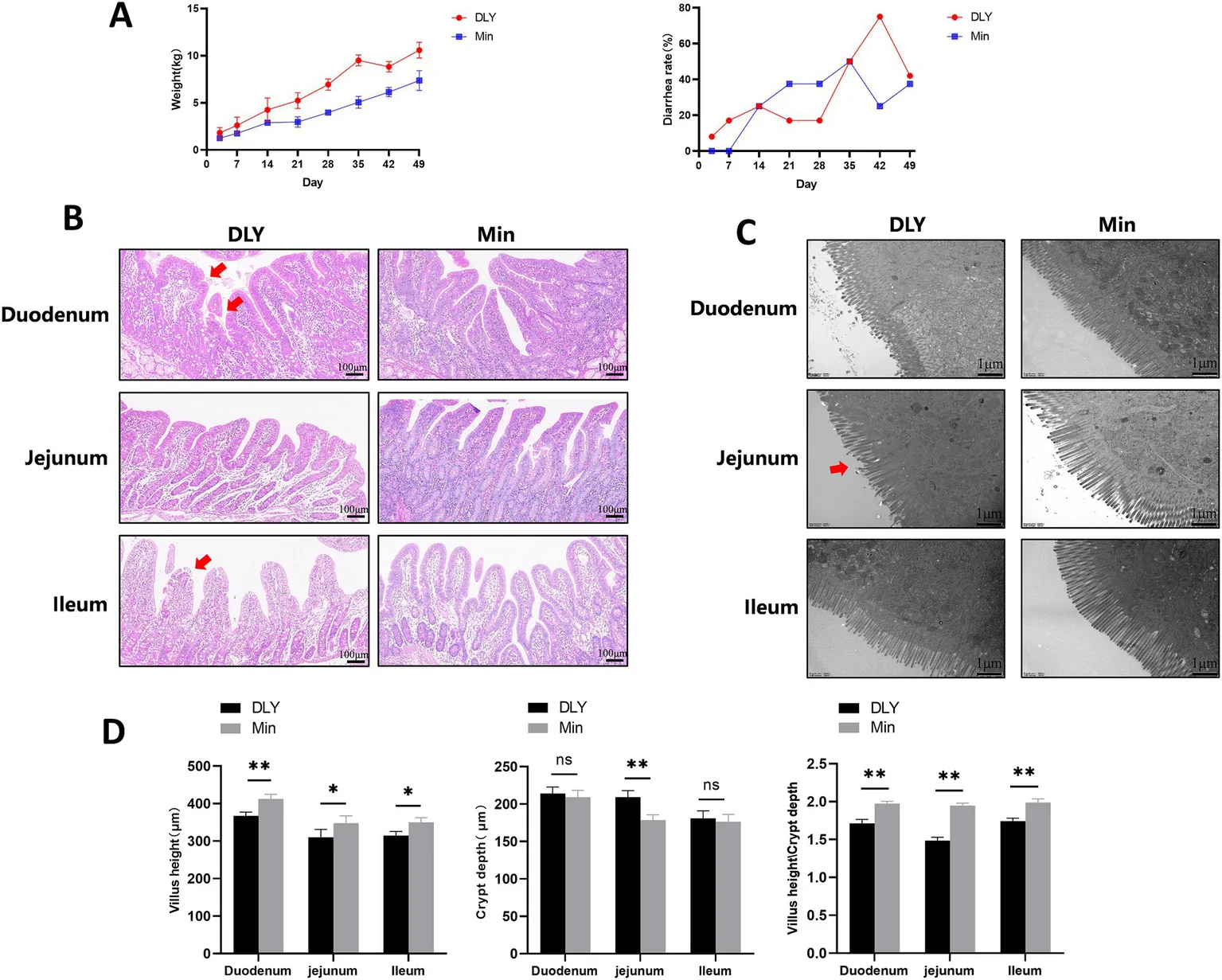
Analysis of the difference in intestinal mucosal barrier function between Min pigs and DLY pigs after weaning. **(A)** Growth performance and diarrhea rate. **(B)** Histological sections of the small intestine from weaned piglets (200×). Arrows indicate representative morphological changes, including irregular villus architecture and epithelial sloughing in DLY pigs, whereas Min pigs showed relatively intact and well-organized villi. **(C)** Representative scanning electron microscopy images showing the ultrastructure of the intestinal epithelial surface in the duodenum, jejunum, and ileum (20,000×). Arrows indicate representative differences in brush-border/microvillar organization between DLY and Min pigs. **(D)** Determination of small intestinal villi and crypts in piglets. Statistical significance denoted as follows: *p < 0.05; **p < 0.01; ***p < 0.001; ns *p* > 0.05.

Histological examination using H&E staining and SEM was performed on tissue sections from both groups to assess the inflammatory response and lesions. Histological examination showed that the duodenum, jejunum, and ileum of Min pigs maintained relatively intact and well-organized villus architecture, whereas DLY pigs exhibited more irregular villus morphology and occasional epithelial sloughing (Figure 6B). Ultrastructural examination further revealed differences in the organization of the apical brush border between the two breeds (Figure 6C). In addition, Min pigs exhibited greater villus height and crypt depth than DLY pigs (Figure 6D).

The inflammatory response was assessed by measuring the expression of key inflammatory markers (Figure 7A). DLY pigs showed significantly higher levels of inflammatory cytokines, particularly in the ileum, compared to Min pigs, with no significant differences observed in the jejunum. Tight junction integrity was evaluated by detecting the mRNA and protein expression levels of tight junction proteins (Figures 7B,C). Min pigs exhibited significantly higher levels of tight junction proteins across all intestinal segments, suggesting better gut barrier integrity compared to DLY pigs. Toll-like receptors (TLRs), a crucial type of pattern recognition receptors (PRRs), are extensively found on the surface or inside different cell types and are vital for microbial detection by the innate immune system(Kawai and Akira, 2011). The DLY group exhibited significantly higher expression levels of TLR4 and NF-κB in the duodenum compared to the Min group. In the jejunum, TLR4 expression was significantly elevated in the DLY group compared to the Min group. Similarly, in the ileum, the DLY group exhibited significantly higher expression levels of TLR4, MyD88, and NF-κB than the Min group (Figure 7D).

**Figure 7:**
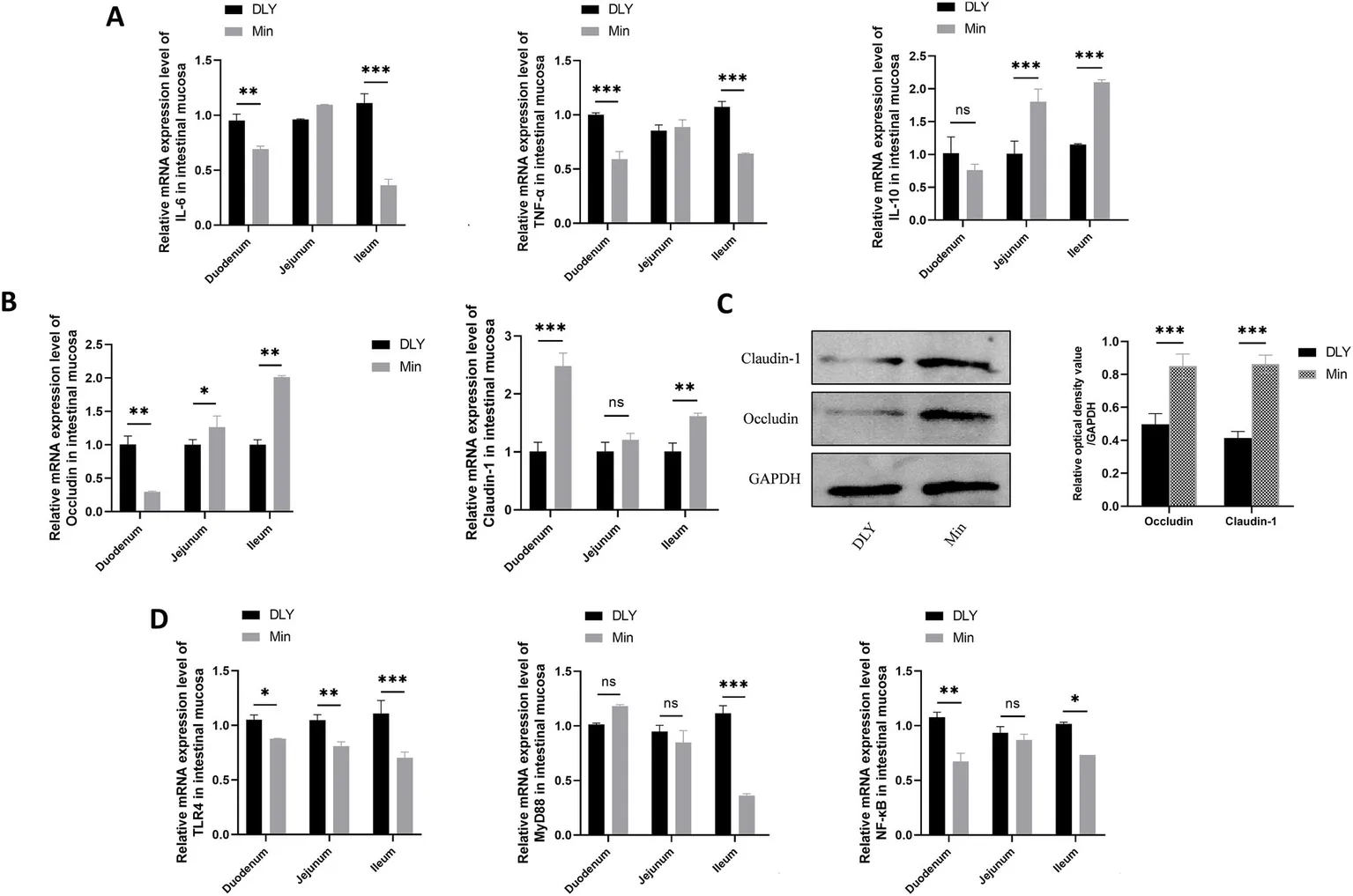
Differences in inflammatory responses, gut barrier integrity, and TLR4/NF-κB signaling between Min and DLY pigs. **(A)** Relative mRNA expression of inflammatory cytokines IL-6, TNF-α, and IL-10. **(B)** Relative mRNA expression of tight-junction genes *Occludin and Claudin-1*. **(C)** Relative expression levels of *Claudin-1* and *Occludin* proteins in the ileal mucosal tight junction. **(D)** Relative mRNA expression of TLR4, MyD88, and NF-κB. Statistical significance denoted as follows: *p < 0.05; **p < 0.01; ***p < 0.001; ns *p* > 0.05.

### 3.7 FMT remodels DLY pig microbiota toward healthier profiles

The overall study design is illustrated in Figure 8A. Alpha diversity analysis using chao1 and Shannon indices indicated that microbial richness and diversity were greater in the FMT group compared to the control group on both day 28 and day 42, implying that FMT improved microbial diversity in DLY pigs (Figures 8B,C).

**Figure 8:**
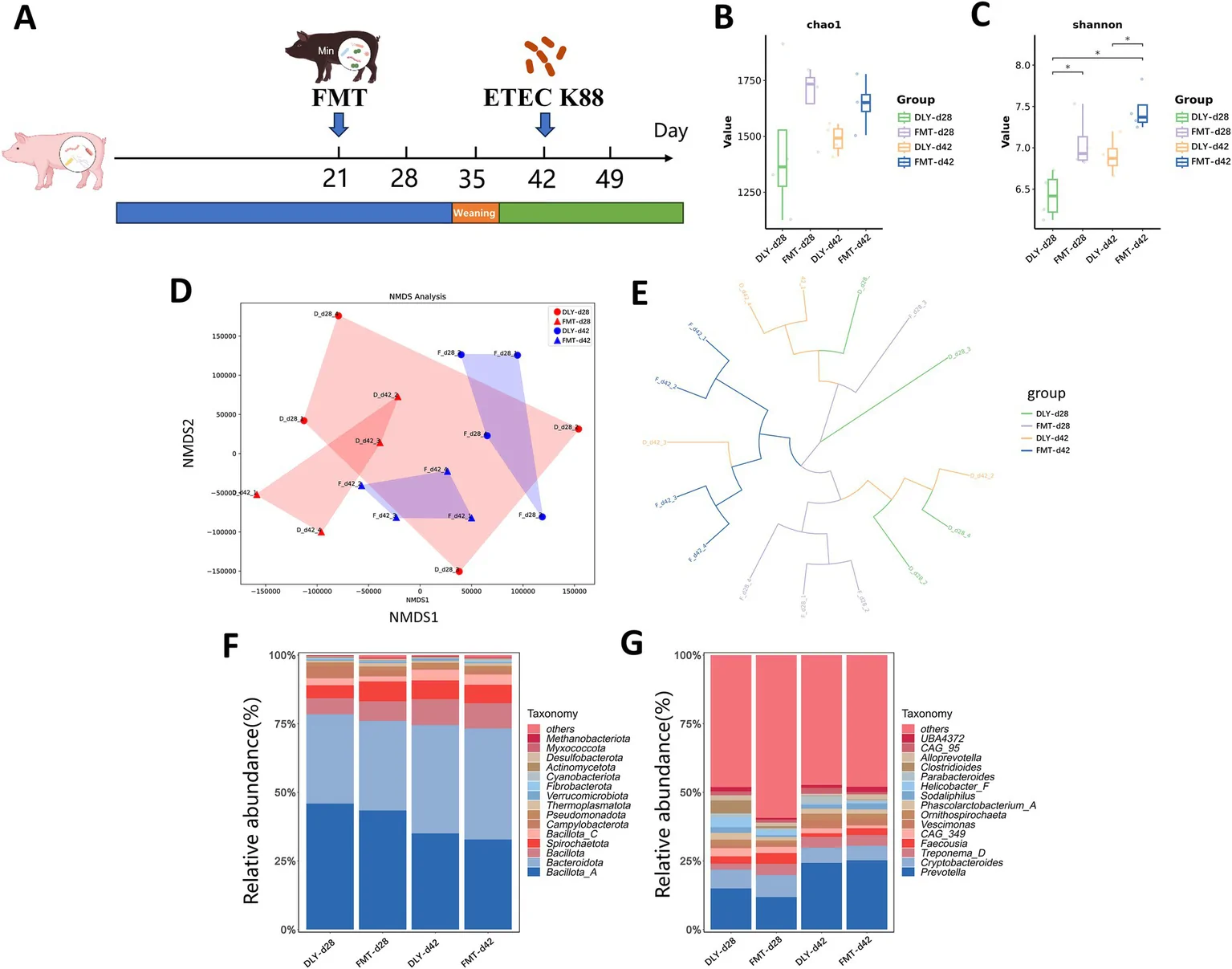
Effects of fecal microbiota transplantation on gut microbiota of pigs. **(A)** Experimental flow of the *in vivo* experiment. **(B)** Chao1 index showing alpha diversity. **(C)** Shannon index showing alpha diversity. **(D)** β-diversity analysis using NMDS ordination based on Bray-Curtis distances. **(E)** β-diversity analysis using UPGMA clustering based on unweighted UniFrac phylogenetic distances. **(F)** Effect of fecal microbiota transplantation at the phylum level. **(G)** Effect of fecal microbiota transplantation at the genus level.

NMDS analysis based on binary Jaccard distance indicated a distinct separation between the gut microbial communities of the FMT and CON groups (Figure 8D). This was further supported by Unweighted Pair Group Method with Arithmetic Mean (UPGMA) clustering, where FMT and control samples formed distinct clusters with higher intra-group similarity and greater inter-group differences, indicating that FMT significantly altered the gut microbiota structure (Figure 8E).

The relative abundance of gut microbiota at the phylum and genus levels is presented in Figures 8F,G. *Bacteroidota*, *Bacillota*, and Spirochaetota were the predominant phyla in both groups. FMT led to a higher relative abundance of *Bacteroidota* and a lower abundance of *Bacillota* on both day 28 and day 42. At the genus level, *Faecousia* showed increased abundance in the FMT group. On day 28, the FMT group showed an increase in *Vescimonas* and *Cryptobacteroides*, while Clostridioides significantly decreased in relative abundance.

### 3.8 FMT repairs ETEC-induced gut damage via barrier-immune crosstalk

Compared with the ETEC group, the FMT group showed a significant improvement in pig weight over time, alleviating the growth retardation caused by ETEC K88 infection. The FMT group exhibited a significantly lower incidence of diarrheal pigs compared with the ETEC group, demonstrating that FMT effectively mitigated ETEC-induced diarrhea and facilitated weight gain in recipient pigs (Figures 9A,B). Pre-treatment with fecal microbiota significantly reduced ETEC K88 colonization in the intestinal tract (Figure 9C). The FMT group showed notably elevated serum ETEC-specific IgG and secretory IgA (SIgA) levels in the jejunal and ileal mucosa compared to the ETEC group, while the control and ETEC groups did not differ significantly (Figure 9D).

**Figure 9:**
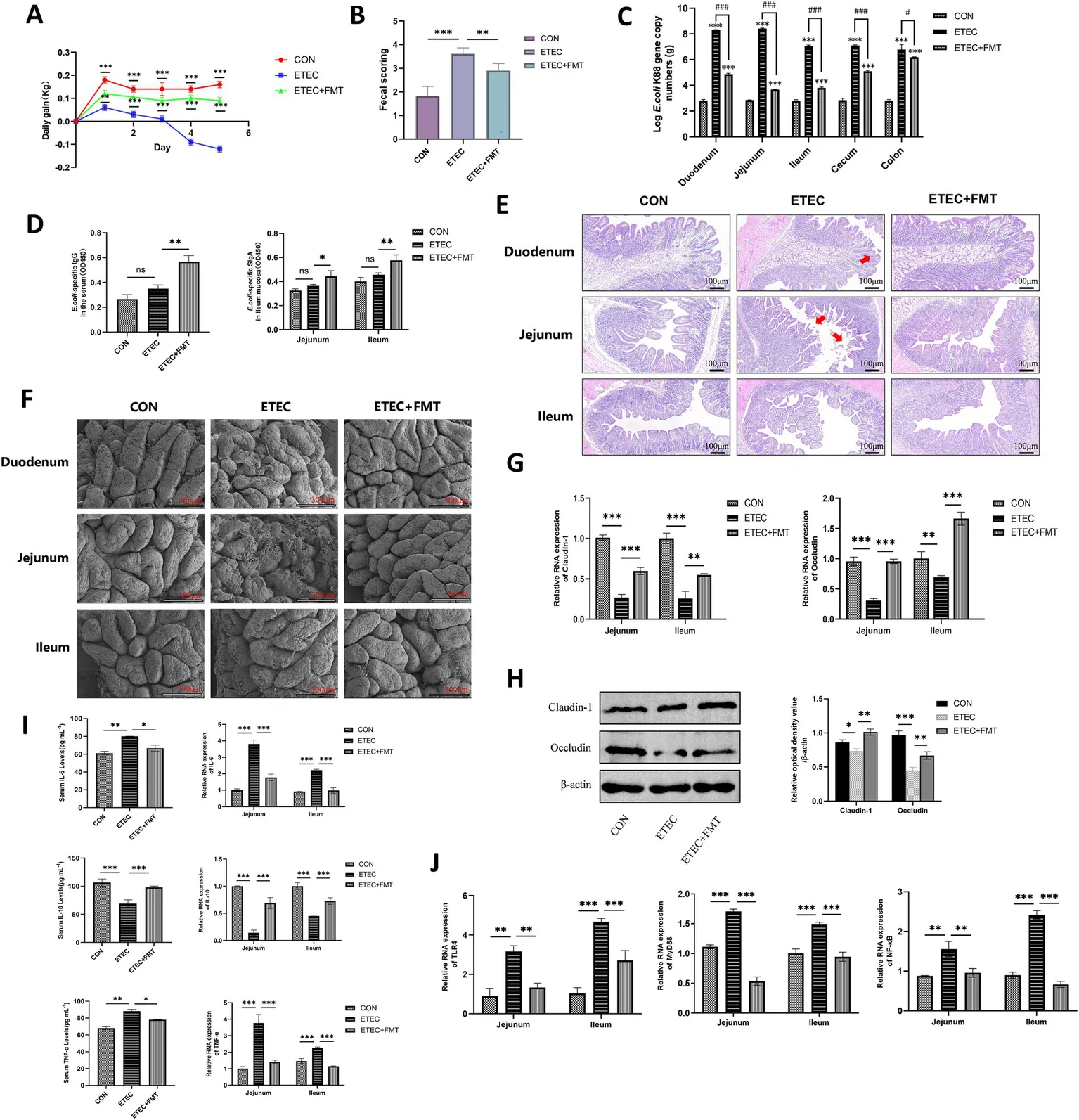
FMT improves intestinal health and immune responses in ETEC-challenged pigs. **(A)** Effects of FMT on growth performance of piglets after infection. **(B)** Fecal scores of piglets following ETEC challenge. **(C)** ETEC K88 loads in different intestinal segments. **(D)** Effects of FMT on serum ETEC-specific IgG and intestinal SIgA levels. **(E)** Representative H&E-stained sections of the duodenum, jejunum, and ileum from CON, ETEC, and ETEC+FMT piglets (200×). Red arrows indicate representative areas of disrupted or irregular villus architecture in the ETEC group. **(F)** Effect of FMT on the intestinal ultrastructure of piglets after infection as observed by scanning electron microscopy (300×). **(G)** Relative mRNA expression of the tight-junction genes *Claudin-1* and *Occludin* in the jejunum and ileum. **(H)** Protein expression of Claudin-1 and Occludin in intestinal tissues. **(I)** The effects of FMT on serum secretion levels and mRNA expression of the inflammatory cytokines IL-6, IL-10, and TNF-α after infection. **(J)** Expression of TLR4 signaling pathway related genes in infected. Statistical significance is denoted as follows: *p < 0.05; **p < 0.01; ***p < 0.001 vs. CON; ^#^*p* < 0.05, ^##^*p* < 0.01, ^###^*p* < 0.001; vs. ETEC.

Histological examination using H&E staining and SEM indicated significant villous damage in the duodenum, jejunum, and ileum of pigs from the ETEC group. In contrast, intestinal morphology in the FMT group was markedly preserved, with significantly reduced villous damage (Figures 9E,F). FMT restored the expression of tight junction proteins Occludin and Claudin-1 at both mRNA and protein levels, mitigating ETEC-induced cytotoxicity *in vivo* (Figures 9G,H). The FMT group exhibited significantly reduced expression levels of inflammatory cytokines IL-10, IL-6, and TNF-α in both serum and colonic tissues compared to the ETEC group (Figure 9I). Activation of TLR4 through the MyD88-dependent pathway triggers NF-κB and promotes pro-inflammatory cytokine production. Our study found that ETEC K88 infection elevated TLR4, MyD88, and NF-κB at mRNA levels in the ileum of weaned pigs, whereas these levels were markedly reduced in the FMT group (Figure 9J).

Overall, these findings suggest that ETEC K88 compromises intestinal barrier integrity and increases intestinal permeability, whereas FMT treatment restores barrier function and mitigates epithelial damage and inflammation.

## 4 Discussion

The trade-off between shortened production cycles through early weaning and its consequences (increased diarrhea, mortality, and costs) is particularly pronounced in commercial versus indigenous pig breeds (Gresse et al., 2017). The gut microbiota is essential for host health, with changes in its composition and abundance linked to the development of various chronic diseases (El Aidy et al., 2015). This study characterized the temporal dynamics of porcine gut microbiota across eight developmental time points, revealing spatiotemporal distribution patterns and identifying microbiota shifts in all intestinal segments by day 49. Animal gut microbiota experiences temporal and spatial variations, significantly contributing to the formation of a stable microecosystem (Huang et al., 2020). A stable microbiota is crucial for animal health, with its composition affected by factors such as species, diet, age, and health status (Han et al., 2018). Distinct microbiota profiles require tailored nutritional strategies and breeding approaches, and can indicate nutritional disorders such as diarrhea in pigs (Yang et al., 2021).

As pigs aged, both alpha diversity and microbial richness showed an overall increasing trend, consistent with previous studies (Lu et al., 2018; Niu et al., 2015). Based on fecal microbiota diversity patterns, pigs were categorized into three developmental stages: immediate post-farrowing (3 days old), pre-weaning (7–35 days old), and post-weaning. Research consistently identifies *Bacillota* and *Bacteroidota* as the primary phyla in mammalian gut microbiota (Arsenault et al., 2024). In line with Yang et al., who observed an increase in Bacillota with age and a higher abundance of Bacteroidota in younger pigs, our results confirmed that the gut microbiota of both DLY and Min pigs was primarily composed of these two phyla (Yang et al., 2021). The differences in disease resistance between breeds may be attributed to distinct intestinal microenvironments or microbial interactions, including bacteria-host dynamics, which may modulate immune responses and contribute to breed-specific resilience (Yoo et al., 2020). *Prevotella* sp000434975, *Phocaeicola vulgatus*, and *Bacteroides fragilis* were the predominant species in the intestinal tracts of both Min and DLY pigs. *Prevotella* is able to assist animals in the breakdown of complex organic matter such as cellulose (Chen et al., 2021; Tett et al., 2021). Given the rapid growth of DLY pigs, which require efficient feed digestion and nutrient absorption, the increased abundance of Prevotella may enhance their ability to digest certain feed components and meet the energy demands of rapid growth (Hu et al., 2023). Chen et al. discovered a significant link between *Prevotella* and fat accumulation in pigs, suggesting that this bacterium’s colonization might influence the expression of genes involved in fat metabolism via specific signaling pathways (Chen et al., 2021). The intestinal tracts of Min pigs exhibited a higher overall relative abundance of *Phocaeicola-vulgatus* and *Bacteroides*-fragilis compared to DLY pigs. *Phocaeicola-vulgatus* significantly alleviates DSS-induced ulcerative colitis and reduces both animal and cellular inflammation levels, highlighting its crucial role in anti-inflammation and immunomodulation (Mills et al., 2022). The metabolite 3-phenylpropanoic acid, produced by *Bacteroides fragilis*, enhances intestinal epithelial barrier function through activation of the aryl hydrocarbon receptor (AhR) signaling pathway (Hu et al., 2024).

Our study revealed that Min pigs exhibited a higher relative abundance of probiotic bacteria, specifically *Lactobacillus amylovorus* and *Lactobacillus johnsonii*, in their intestinal segments on day 49 compared to DLY pigs. This observation is consistent with previous reports linking these *Lactobacillus* species to enhanced resilience to stress and inflammation (Jia et al., 2022; Shen et al., 2022). These breed-associated microbial differences were accompanied by distinct intestinal mucosal phenotypes. Compared with DLY pigs, Min pigs showed better-preserved villus architecture, greater villus height, higher expression of the tight-junction proteins Claudin-1 and Occludin, and lower expression of several inflammatory signaling components. Greater villus height may reflect a larger absorptive surface, whereas differences in crypt depth may indicate altered epithelial renewal and turnover. Together, these findings suggest that the breed-specific gut microbiota of Min pigs is associated with better maintenance of intestinal barrier integrity and a more balanced inflammatory state after weaning.

Given the observed microbial differences between DLY and Min pigs, we further investigated whether microbiota transplantation could confer protective effects against pathogen challenge. Our results showed that FMT altered the gut microbial composition in DLY pigs and enhanced early-life resistance to enteric infection.

ETEC, particularly the K88 strain, is a major diarrheagenic pathogen in both humans and animals. Pigs are typically weaned at 3–4 weeks of age, a critical period when they are especially vulnerable to infection. Although vaccines are available, their protective efficacy against post-weaning ETEC infection remains limited (Sun and Kim, 2017), possibly due to the loss of maternally derived antibodies. The decline in intestinal antibody levels following weaning compromises mucosal immunity, increasing susceptibility to ETEC colonization (Dubreuil, 2021). ETEC K88 infection is known to induce intestinal inflammation and diarrhea by disrupting the epithelial barrier and upregulating pro-inflammatory cytokines (Ma et al., 2025). Infection reduces tight junction protein expression, including ZO-1 and Claudin-1, in the ileum of neonatal pigs. Previous studies have shown that FMT mitigates inflammation and preserves epithelial integrity in pigs challenged with lipopolysaccharide (Geng et al., 2018), and promotes mucosal protective autophagy while reducing ETEC-induced damage (Cheng et al., 2018). Our study confirmed that ETEC K88 infection increased pro-inflammatory cytokines and decreased tight junction protein expression, consistent with previous reports (Cheng et al., 2018; Qiao et al., 2020; Xiang et al., 2020). The villus disruption and epithelial sloughing observed after ETEC challenge indicate mucosal structural injury, whereas the preserved villus morphology in the FMT group suggests reduced epithelial damage. FMT also significantly reduced ETEC K88 bacterial loads in intestinal segments by day 49, evidenced by the absence of rod-shaped bacterial adherence in treated pigs. ETEC infection impairs the mucosal barrier by reducing MUC2 expression, either through goblet cell damage or direct degradation of the protein (Sheikh et al., 2022). Our findings show that FMT enhanced *MUC2* expression and increased the levels of ZO-1, Claudin-1, and Occludin, supporting previous research indicating that FMT strengthens the intestinal barrier (Ma X. et al., 2021; Sampath et al., 2024). Claudin-1 and Occludin are major components of epithelial tight junctions that restrict paracellular permeability and maintain mucosal barrier integrity. Their reduced expression following ETEC challenge is therefore consistent with barrier disruption, whereas their restoration after FMT supports preservation of epithelial integrity. These results suggest that FMT improves intercellular junctions, strengthens mucosal integrity, and reduces ETEC colonization (Ma T. et al., 2021). Notably, DLY pigs receiving fecal transplants from healthy Min pigs exhibited disease resistance comparable to indigenous Min pigs. Cytokines like TNF-α, IL-6, and IL-10 play pivotal roles in inflammation regulation, with TNF-α and IL-6 promoting inflammation and IL-10 acting as an anti-inflammatory agent (Ma X. et al., 2021; Popivanova et al., 2008). TLR4 signals through the adaptor MyD88 to activate NF-κB-mediated inflammatory responses. In our study, FMT significantly downregulated TLR4, MyD88, and NF-κB mRNA expression in the jejunum and ileum of infected pigs. This reduction was accompanied by lower TNF-α and IL-6 levels, consistent with attenuation of ETEC-associated inflammatory activation (Sun, 2017).

The study indicates that ETEC K88 infection triggers the MyD88-dependent TLR4/MyD88/NF-κB signaling pathway in pigs, influencing immune responses. Increased TLR4 and MyD88 expression may activate NF-κB, affecting the expression of anti-inflammatory cytokines like IL-10 and pro-inflammatory cytokines such as TNF-α and IL-6. The FMT group showed significantly lower mRNA expression levels of TLR4, MyD88, and NF-κB compared to the infected group, but these levels were still higher than those in the control group. These results imply that FMT may help restore immune homeostasis by modulating the MyD88-dependent pathway, thereby enhancing the host’s innate immune defense against ETEC K88. Although the number of animals in the breed-comparison and FMT/ETEC experiments was limited, these *in vivo* experiments were conducted to obtain initial evidence for breed-associated intestinal differences and the protective effects of FMT while minimizing animal use in accordance with animal welfare principles. Further studies with larger, independent cohorts are required to confirm the reproducibility and broader applicability of these findings.

## 5 Conclusion

This study demonstrates that gut microbiota development in pigs is strongly shaped by age, intestinal segment, and host breed. Weaning represents a critical transition point characterized by transient reductions in microbial diversity and pronounced community restructuring, while distinct microbial profiles are maintained between the small and large intestines. Comparative analyses revealed marked breed-specific differences in microbial succession. Compared with DLY pigs, Min pigs exhibited higher bacterial and fungal diversity, greater enrichment of probiotic-associated taxa, and enhanced predicted metabolic capacity, particularly during early life and post-weaning stages. Additionally, Min pigs displayed stronger intestinal barrier function via enhanced mucosal defenses. FMT improved the microbial diversity of commercial DLY pigs and their resistance to ETEC K88.

## Data Availability

The data analyzed in this study are available in the (https://www.ncbi.nlm.nih.gov) under BioProject accession number PRJNA1276682. Additional study data are included in the article and its Supplementary material.
