# Supplementary material for: Gut microbiota developmental trajectories in min and DLY pigs during lactation and nursery stages and the alleviation of ETEC-induced diarrhea by fecal microbiota transplantation
Source: Front Microbiol. 2026 Oct 26;17:1939230. doi: 10.3389/fmicb.2026.1939230 (PMC13745252; doi:10.3389/fmicb.2026.1939230)
Supplement: Supplementary file 1 [file Supplementary_file_1.DOCX]

**Table S1** Composition and nutrient levels of diet.

| **Ingredients** | **Content** | **Nutrient Levels** | **Content** |
| --- | --- | --- | --- |
| Corn | 58.60 | Digestible energy (MJ/kg) | 13.68 |
| Soybean meal | 17.50 | Crude protein | 20.40 |
| Expanded soybean | 7.50 | Lys | 1.30 |
| Milk powder | 4.00 | Met + Cys | 0.78 |
| Fish meal | 4.00 | Thr | 0.87 |
| Whey powder | 4.30 | Na | 0.27 |
| NaCl | 0.30 | Ca | 0.95 |
| Limestone | 1.22 | Available P | 0.39 |
| CaHPO4 | 1.16 |  |  |
| DL-met | 0.07 |  |  |
| Lys HCL | 0.35 |  |  |
| Premix | 1.00 |  |  |
| Total | 100.00 |  |  |

**Table S2** Primer sequences of cytokines.

| Gene | Primer sequence (5’-3’) | Accession number |
| --- | --- | --- |
| β-actin | F: TGCTGTGTTCCCATCTATCG | AY550069.1 |
|  | R: TTGGTGACAATACCGTGTTCA |  |
| ZO-1 | F: CTGTGTGGGGCCTGACAA | NC_010443.5 |
|  | R: AGTGCTTGCAGTCGAACTCA |  |
| Claudin-1 | F: ATGCCAGAGTAAATGTAC | NC_010455.5 |
|  | R: CCACGTAGTAGACGATGGGC |  |
| TLR4 | F: CCAGAGGTCCGACCACTACA | NC_010443.5 |
|  | R: GGTCCCCTTCAATCCTGTTGAA |  |
| Myd88 | F: CAGCCCAGTTT'GGTCTGTT | NC_010455.5 |
|  | R: TTTCCACTCGGGCTCAATCC |  |
| NF-κB | F: AATGGTTCGGTCGATATCG | NC_010450.4 |
|  | R: TACTGGCGTAGCAAATGC |  |
| GAPDH | F: GCACCCAGCAACGACAT | NC_010447.5 |
|  | R: TCGCGACTCCAAACTCCTTC |  |
| TNF-α | F: GCCCTTCCACCAACGTTTTC | NC_010449.5 |
|  | R: TCCCAGGTAGATGGGTTCGT |  |
| IL-6 | F: ATGCCAGAGTAAATGTAC  R: CCACGTAGTAGACGATGGGC | NC_010451.4 |
| IL-10 | F: CCAGAGGTCCGACCACTACA | NC_010451.4 |
|  | R: GGTCCCCTTCAATCCTGTTGAA |  |
| F4^+^ETEC | F: GCTACAAATGCCTATGCATCTACACA | NR_024570.1 |
|  | R: CATGCTCCAGCAGTACCATCTCTAAC |  |

**Supplementary material S1** Indicator species analysis.

**Supplementary material S2** Species composition of endophytic fungal communities at the phylum and genus level at different days of age. (A) Composition of intestinal fungal communities in DLY pigs at phylum and genus levels. (B) Composition of intestinal fungal communities in Min pigs at phylum and genus levels.

**Supplementary material S3** Co-occurrence network analysis of bacterial community modules on SparCC method.

**Supplementary material S4** Species composition of endophytic fungal communities at the phylum and genus level of different intestinal segments. (A) Composition of intestinal fungal communities in DLY pigs at phylum and genus levels. (B) Composition of intestinal fungal communities in Min pigs at phylum and genus levels.
